# Supplementary material for: Machine learning vs. traditional regression analysis for fluid overload prediction in the ICU
Source: Sci Rep. 2023 Nov 10;13:19654. doi: 10.1038/s41598-023-46735-3 (PMC10638304; doi:10.1038/s41598-023-46735-3)

**Supplementary Material**

**Additional Methods**

**Supplemental Table 1.** Full regression model for presence of fluid overload at 72 hours

**Supplemental Table 2.** Stepwise regression for final model to predict presence of fluid overload

**Supplemental Table 3.** Full regression model for amount of fluid overload at 72 hours

**Supplemental Table 4.** Stepwise regression for final model to predict fluid overload amount

**Supplemental Table 5.** Performance of fluid overload prediction models for amount of fluid overload

**Supplemental Table 6**. Correlation matrix between continuous variables from backward selection and variables from Random Forest model.

**Supplemental Table 7.** Correlation matrix between categorical variables from backward selection and variables from Random Forest model.

**Supplemental Table 8.** Prediction results of backward selected regression with high SOFA and high APACHE II

**Supplemental Figure 1.** Consort diagram

**Supplemental Figure 2.** AUROC curve for fluid overload at 72 hours

**Supplemental Figure 3.** Most common features for presence of fluid overload prediction with Random Forest imputations

**Supplemental Figure 4.** Most common features for presence of fluid overload prediction with Support Vector Machine imputations

**Supplemental Figure 5.** Most common features for amount of fluid overload prediction with XGBoost imputations

**Supplemental Figure 6.** Most common features for amount of fluid overload prediction with Random Forest imputations

**Supplemental Figure 7.** Most common features for amount of fluid overload prediction with Support Vector Machine imputations

**Additional Methods**

*Data missingness:* Variables were excluded if the corresponding missing proportions were over 30% prior to imputation, with the exception of SOFA score. For continuous variables [including APACHE II, SOFA, fluid balance (mL), and amount of fluid overload], linear regression was applied for imputation. During the imputation process, logistic regression was used for binary variables and polytomous logistic regression was used for multi-level variables. Descriptive statistics on the data before multiple imputation were calculated, and clinical characteristics between those patients with and without fluid overload were compared using either Student *t* test or Chi-square test, as appropriate. A p-value of less than 0.05 was considered statistically significant.

*Amount of fluid overload analysis:* All methodology for calculating percent fluid overload as a continuous variable was similar except for appropriate changes to manage a continuous variable including the use of linear regression (instead of logistic) and the use of Random Forest regression, SVM regression, and XGBoost regression instead of Random Forest classifier, SVM classifier, and XGBoost classifier. Finally, performances were measured using mean squared error (MSE) calculated on each imputed testing set and then averaged as the final MSE. To compare performance of the models, a series of values were calculated including for the models with classification task: area under the receiver operating characteristic (AUROC), positive predictive value (PPV), negative predictive value (NPV), specificity, and sensitivity. For regression models, mean square error (MSE) was used to measure the model performance.

*Correlation analysis between Random Forest Model and backward selection variables:* We compared the variables selected from backward selection and top five variables selected from the Random Forest model. Backward selection provided six variables, among which four were not in the variable list selected from Random Forest. These variables were sex-male, admission diagnosis-sepsis/septic shock, laboratory values-serum bicarbonate, and age - ≥ 65). On the other hand, three variables in the top five variable list from the Random Forest model were not selected from backward selection process. These variables were MRC-ICU score at 24 hours, number of continuous IV infusions, and fluid balance at 24 hr (mL). To evaluate the relationship among these variables from two groups, we conducted:

1. Pearson correlation analyses was conducted between APACHE II at 24 hours and SOFA at 24 hours (the two continuous variables from backward selection) AND APACHE II at 24 hours, SOFA at 24 hours, MRC-ICU at 24 hours, number of continuous IV infusions, fluid balance at 24 hr (mL) (the five continuous variables from the Random Forest model). All analyses were based on the multiple imputed data sets.
2. ANOVA analyses was conducted between sex-male, admission diagnosis-sepsis/septic shock, laboratory values-bicarbonate, age - ≥ 65) (the four categorical variables from backward selection) AND APACHE II at 24 hours, SOFA at 24 hours, MRC-ICU at 24 hours, number of continuous IV infusions, fluid balance at 24 hr (mL) (the five continuous variables from the Random Forest model). All analyses were based on multiple imputed data sets.

*Evaluation of the results in those patients with high APACHE-2 and high SOFA scores:* For this analysis we defined a high APACHE-2 as a score ≥25 and high SOFA as a score ≥10). We generated predictions using the backward section model, since it is the simplest model with the greatest explanatory power. These prediction results are contingent on several additional variables, including sex-male, admission diagnosis-sepsis/septic shock, laboratory values-serum bicarbonate, and age = ≥ 65). Consequently, we computed predictions for each combination of these variables in conjunction with high APACHE II score and high SOFA score.

**Supplemental Table 1.** Full regression model for presence of fluid overload at 72 hours

|  | **Univariate** | | | **Multivariate** | | |
| --- | --- | --- | --- | --- | --- | --- |
| **Variable** | **Odds Ratio** | **95% CI** | **p-value** | **Odds Ratio** | **95% CI** | **p-value** |
| **ICU Baseline** | | | | | | |
| Age ≥ 65 years old; mean (SD) | 0.73 | 0.45, 1.18 | 0.19 | 0.57 | 0.29, 1.10 | 0.09 |
| Sex (male) | 0.51 | 0.31, 0.83 | 0.00 | 0.48 | 0.26, 0.88 | 0.01 |
| *Relevant chronic conditions* | | | | | | |
| Chronic kidney disease | 0.42 | 0.05, 3.07 | 0.39 | 0.26 | 0.02, 3.07 | 0.28 |
| Heart failure | 0.80 | 0.18, 3.55 | 0.77 | 3.05 | 0.53, 17.55 | 0.20 |
| Admission to medical ICU | 1.17 | 0.69, 1.97 | 0.53 | 0.92 | 0.41, 2.07 | 0.84 |
| *Primary ICU Admission Diagnosis* | | | | | | |
| Cardiac | 0.42 | 0.16, 1.07 | 0.07 | 0.65 | 0.18, 2.28 | 0.49 |
| Chronic kidney disease | 0.42 | 0.05, 3.07 | 0.39 | 0.26 | 0.02, 3.07 | 0.28 |
| Hepatic | 1.05 | 0.14, 7.86 | 0.95 | 0.43 | 0.02, 6.59 | 0.54 |
| Pulmonary | 1.12 | 0.56, 2.24 | 0.73 | 1.20 | 0.47, 3.06 | 0.68 |
| Sepsis/septic shock | 4.22 | 2.17, 8.21 | 0.00 | 3.50 | 1.51, 8.11 | 0.00 |
| Trauma | 1.19 | 0.41, 3.40 | 0.75 | 0.46 | 0.11, 1.98 | 0.30 |
| **24 hours after ICU admission** | | | | | | |
| *Severity of illness, using worst values recorded* | | | | | | |
| APACHE II Score | 1.11 | 1.07, 1.15 | 0.00 | 1.07 | 0.99, 1.16 | 0.06 |
| SOFA Score | 1.31 | 1.21, 1.42 | 0.00 | 1.20 | 0.99, 1.44 | 0.05 |
| *Supportive care devices* | | | | | | |
| Renal replacement therapy | 0.85 | 0.10, 6.79 | 0.88 | 0.17 | 0.01, 2.00 | 0.16 |
| Invasive mechanical ventilation | 2.95 | 1.89, 4.61 | 0.00 | 0.67 | 0.25, 1.77 | 0.42 |
| *Laboratory values (serum) and flowsheet values* | | | | | | |
| Bicarbonate < 22 mEq/L | 0.37 | 0.22, 0.62 | 0.00 | 0.59 | 0.31, 1.13 | 0.11 |
| Bicarbonate > 29 mEq/L | 0.22 | 0.09, 0.53 | 0.00 | 0.36 | 0.12, 1.10 | 0.07 |
| Creatinine ≥ 1.5 mg/dL | 2.48 | 1.25, 4.91 | 0.00 | 0.99 | 0.27, 3.59 | 0.98 |
| Chloride ≥ 110 mEq/L |  |  |  |  |  |  |
| Potassium ≥ 5.5 mEq/L | 2.39 | 0.96, 5.90 | 0.05 | 1.25 | 0.38, 4.12 | 0.70 |
| Sodium ≥ 148 mEq/L | 3.08 | 1.42, 6.68 | 0.00 | 1.53 | 0.53, 4.36 | 0.42 |
| Sodium <134 mEq/L | 0.68 | 0.26, 1.74 | 0.42 | 0.35 | 0.11, 1.08 | 0.06 |
| Fluid balance (mL) | 1.35 | 1.14, 1.61 | 0.00 | 1.15 | 0.87, 1.50 | 0.28 |
| Acute kidney injury | 3.21 | 1.93, 5.33 | 0.00 | 1.72 | 0.72, 4.11 | 0.22 |
| *Medications* | | | | | | |
| MRC-ICU mean (SD) | 1.06 | 1.04, 1.09 | 0.00 | 1.05 | 0.98, 1.13 | 0.14 |
| Vasopressor use in first 24 hours | 2.24 | 1.37, 3.67 | 0.00 | 0.60 | 0.24, 1.47 | 0.26 |
| Use of continuous infusions | 2.04 | 1.25, 3.33 | 0.00 | 1.07 | 0.50, 2.31 | 0.84 |
| Number of continuous infusions | 1.05 | 0.98, 1.13 | 0.14 | 0.87 | 0.73, 1.04 | 0.14 |
| *Data are presented as n (%) or mean ± standard deviation (SD) unless otherwise stated.*  *Albumin and lactate are not presented due to missingness exceeding 30%.* | | | | | | |

**Supplemental Table 2.** Stepwise regression for final model to predict presence of fluid overload

| **Variable** | **Odds Ratio** | **95% Confidence Interval** | **p-value** |
| --- | --- | --- | --- |
| Admission Diagnosis-Sepsis/septic shock | 3.52 | 1.64, 7.54 | 0.00 |
| Male | 0.44 | 0.25, 0.77 | 0.00 |
| SOFA at 24 hours | 1.31 | 1.20, 1.42 | 0.00 |
| Sodium ≥ 148 mEq/L | 2.11 | 0.87, 5.11 | 0.09 |
| Sodium <134 mEq/L | 0.41 | 0.14, 1.13 | 0.08 |
| Bicarbonate < 22 mEq/L | 0.51 | 0.29, 0.90 | 0.02 |
| Bicarbonate > 29 mEq/L | 0.29 | 0.11, 0.77 | 0.01 |
| SOFA-Sequential Organ Failure Assessment, OR- odds ratio, CI- confidence interval | | | |

**Supplemental Table 3.** Full regression model for amount of fluid overload at 72 hours

|  | **Univariate** | | | | **Multivariate** | | |
| --- | --- | --- | --- | --- | --- | --- | --- |
| **Variable** | **Odds Ratio** | **95% CI** | **p-value** | | **Odds Ratio** | **95% CI** | **p-value** |
| **ICU Baseline** | | | | | | | |
| Age ≥ 65 years old; mean (SD) | 0.00 | -0.01, 0.00 | | 0.08 | 0.00 | -0.01, 0.00 | 0.02 |
| Sex (male) | -0.01 | -0.01, 0.00 | | 0.00 | 0.00 | -0.01, 0.00 | 0.00 |
| *Relevant chronic conditions* | | | | | | | |
| Chronic kidney disease | -0.00 | -0.03, 0.01 | | 0.50 | -0.01 | -0.03, 0.00 | 0.22 |
| Heart failure | -0.00 | -0.0, 0.01 | | 0.62 | 0.01 | 0.00, 0.03 | 0.13 |
| Admission to medical ICU | 0.00 | 0.00, 0.01 | | 0.12 | 0.00 | 0.00, 0.01 | 0.77 |
| *Primary ICU Admission Diagnosis* | | | | | | | |
| Cardiac | -0.01 | -0.02, 0.00 | | 0.00 | -0.01 | -0.02, 0.00 | 0.02 |
| Hepatic | 0.00 | -0.02, 0.03 | | 0.83 | 0.00 | -0.03, 0.0 | 0.46 |
| Pulmonary | 0.00 | 0.00, 0.01 | | 0.59 | 0.00 | -0.01, 0.01 | 0.91 |
| Sepsis/septic shock | 0.03 | 0.01, 0.04 | | 0.00 | 0.01 | 0.00, 0.02 | 0.02 |
| Trauma | 0.00 | -0.01, 0.01 | | 0.82 | -0.01 | -0.03, 0.00 | 0.03 |
| **24 hours after ICU admission** | | | | | | | |
| *Severity of illness, using worst values recorded* | | | | | | | |
| APACHE II Score | 0.00 | 0.00, 0.00 | | 0.00 | 0.00 | 0.00, 0.00 | 0.03 |
| SOFA Score | 0.00 | 0.00, 0.00 | | 0.00 | 0.00 | 0.00, 0.00 | 0.01 |
| *Supportive care devices* | | | | | | | |
| Renal replacement therapy | -0.00 | -0.03, 0.02 | | 0.73 | -0.05 | -0.07, -0.02 | 0.00 |
| Invasive mechanical ventilation | 0.02 | 0.02, 0.03 | | 0.00 | -0.00 | -0.01, 0.00 | 0.17 |
| *Laboratory values (serum) and flowsheet values* | | | | | | | |
| Bicarbonate < 22 mEq/L | -0.02 | -0.03, -0.01 | | 0.00 | -0.00 | -0.01, 0.00 | 0.10 |
| Bicarbonate > 29 mEq/L | -0.03 | -0.04, -0.02 | | 0.00 | -0.01 | -0.02, 0.00 | 0.03 |
| Creatinine ≥ 1.5 mg/dL | 0.02 | 0.01, 0.03 | | 0.00 | 0.0019 | -0.01, 0.02 | 0.84 |
| Chloride ≥ 110 mEq/L | 0.02 | 0.01, 0.03 | | 0.00 | 0.00 | 0.00, 0.01 | 0.21 |
| Potassium ≥ 5.5 mEq/L | 0.02 | 0.00, 0.03 | | 0.01 | 0.00 | -0.01, 0.01 | 0.87 |
| Sodium ≥ 148 mEq/L | 0.03 | 0.02, 0.05 | | 0.00 | 0.0154 | 0.00, 0.031 | 0.05 |
| Sodium <134 mEq/L | 0.00 | -0.01, 0.00 | | 0.66 | -0.01 | -0.02, 0.00 | 0.05 |
| Fluid balance (mL) | 0.00 | 0.00, 0.00 | | 0.00 | 0.00 | 0.00, 0.00 | 0.03 |
| Acute kidney injury | 0.03 | 0.01, 0.04 | | 0.00 | 0.01 | 0.00, 0.02 | 0.05 |
| *Medications* | | | | | | | |
| MRC-ICU mean (SD) | 0.00 | 0.00, 0.00 | | 0.00 | 0.00 | 0.00, 0.00 | 0.01 |
| Vasopressor use in first 24 hours | 0.00 | 0.00, 0.00 | | 0.00 | 0.00 | 0.00, 0.00 | 0.01 |
| Use of continuous infusions | 0.02 | 0.01, 0.02 | | 0.00 | 0.00 | 0.00, 0.01 | 0.13 |
| Number of continuous infusions | 0.00 | 0.00, 0.00 | | 0.00 | 0.00 | 0.00, 0.00 | 0.05 |
| *Data are presented as n (%) or mean ± standard deviation (SD) unless otherwise stated.*  *Albumin and lactate are not presented due to missingness exceeding 30%.* | | | | | | | |

**Supplemental Table 4.** Stepwise regression for final model to predict fluid overload amount

| **Variable** | **Estimate** | **95% CI** | **p-value** |
| --- | --- | --- | --- |
| Fluid balance at 24 hr (mL) | 0.00 | 0.00, 0.00 | 0.01 |
| Sodium ≥ 148 mEq/L | 0.02 | 0.00, 0.03 | 0.00 |
| Sodium <134 mEq/L | -0.00 | -0.02, 0.00 | 0.10 |
| Sex (Male) | -0.00 | -0.01, 0.00 | 0.00 |
| Admission Diagnosis-Trauma | -0.01 | -0.03, 0.00 | 0.02 |
| Admission Diagnosis-Sepsis/septic shock | 0.01 | 0.00, 0.03 | 0.00 |
| Admission Diagnosis-Cardiac | -0.01 | -0.02, 0.00 | 0.00 |
| Age (>=65) | -0.00 | -0.01, 0.00 | 0.00 |
| SOFA at 24 hours | 0.00 | 0.00, 0.00 | 0.00 |
| Bicarbonate < 22 mEq/L | -0.01 | -0.02, 0.00 | 0.06 |
| Bicarbonate > 29 mEq/L | -0.01 | -0.02, 0.00 | 0.00 |
| APACHE II at 24 hours | 0.00 | 0.00, 0.002 | 0.00 |
| APACHE II- Acute Physiology and Chronic Health Evaluation, SOFA- sequential organ failure assessment | | | |

**Supplemental Table 5.** Performance of fluid overload prediction models for amount of fluid overload

| **Variable** | **Full Regression** | **Stepwise Selected Regression** | **MRC-ICU Regression** | **Random Forest** | **Support Vector Machine** | **XGBoost** |
| --- | --- | --- | --- | --- | --- | --- |
| **Mean Squared Error** | 0.002 | 0.002 | 0.002 | 0.001 | 0.002 | 0.002 |

**Supplemental Table 6.** Correlation matrix between continuous variables from backward selection and variables from Random Forest model.

|  | **APACHE II**  **at 24 hours** | **SOFA**  **at 24 hours** | **MRC-ICU**  **at 24 hours** | **Number of**  **continuous IV**  **infusions** | **Fluid balance**  **at 24 hr (mL)** |
| --- | --- | --- | --- | --- | --- |
| **APACHE II**  **at 24 hours** | 1.00 | 0.62 (0.57, 0.65) | 0.62 (0.58, 0.66) | 0.49 (0.44, 0.53) | 0.17 (0.11, 0.23) |
| **SOFA at 24 hours** | 0.61 (0.57, 0.65) | 1.00 | 0.46 (0.41, 0.51) | 0.33 (0.28, 0.39) | 0.39 (0.33, 0.44) |

**Supplemental Table 7.** Correlation matrix between categorical variables from backward selection and variables from Random Forest model.

|  | | | | | |
| --- | --- | --- | --- | --- | --- |
|  | **APACHE-II**  **at 24 hours** | **SOFA**  **at 24 hours** | **MRC-ICU**  **at 24 hours** | **Number of**  **continuous IV infusions** | **Fluid balance**  **at 24 hr (mL)** |
| **Sex-male** | 0.94168 | 0.02945 | 0.04069 | 0.00431 | 0.50699 |
| **Admission diagnosis-sepsis/**  **septic shock** | 0.29997 | 0.00546 | 0.58847 | 0.17804 | 0.03045 |
| **Laboratory values-serum bicarbonate** | 0.00001 | 0.00004 | 0.10875 | 0.40255 | 0.00001 |
| **Age - ≥ 65** | 0.00000 | 0.04400 | 0.30003 | 0.05321 | 0.00069 |

**Supplemental Table 8.** Prediction results of backward selected regression with high SOFA and high APACHE II

| **Sex** | **Admission diagnosis-sepsis/septic shock** | **Serum bicarbonate** | **Age ≥ 65** | **Fluid Overload Prediction** |
| --- | --- | --- | --- | --- |
| Female | No | 22-29 mEq/L | No | Yes |
| Female | No | < 22 mEq/L | No | Yes |
| Female | No | >29 mEq/L | No | Yes |
| Female | No | 22-29 mEq/L | Yes | Yes |
| Female | No | < 22 mEq/L | Yes | Yes |
| Female | No | >29 mEq/L | Yes | Yes |
| Female | Yes | 22-29 mEq/L | No | Yes |
| Female | Yes | < 22 mEq/L | No | Yes |
| Female | Yes | >29 mEq/L | No | Yes |
| Female | Yes | 22-29 mEq/L | Yes | Yes |
| Female | Yes | < 22 mEq/L | Yes | Yes |
| Female | Yes | >29 mEq/L | Yes | Yes |
| Male | No | 22-29 mEq/L | No | Yes |
| Male | No | < 22 mEq/L | No | Yes |
| Male | No | >29 mEq/L | No | Yes |
| Male | No | 22-29 mEq/L | Yes | Yes |
| Male | No | < 22 mEq/L | Yes | Yes |
| Male | No | >29 mEq/L | Yes | No |
| Male | Yes | 22-29 mEq/L | No | Yes |
| Male | Yes | < 22 mEq/L | No | Yes |
| Male | Yes | >29 mEq/L | No | Yes |
| Male | Yes | 22-29 mEq/L | Yes | Yes |
| Male | Yes | < 22 mEq/L | Yes | Yes |
| Male | Yes | >29 mEq/L | Yes | Yes |

**Supplemental Figure 1.** Consort diagram

**Supplemental Figure 2.** AUROC Curve for Fluid Overload at 72 hours


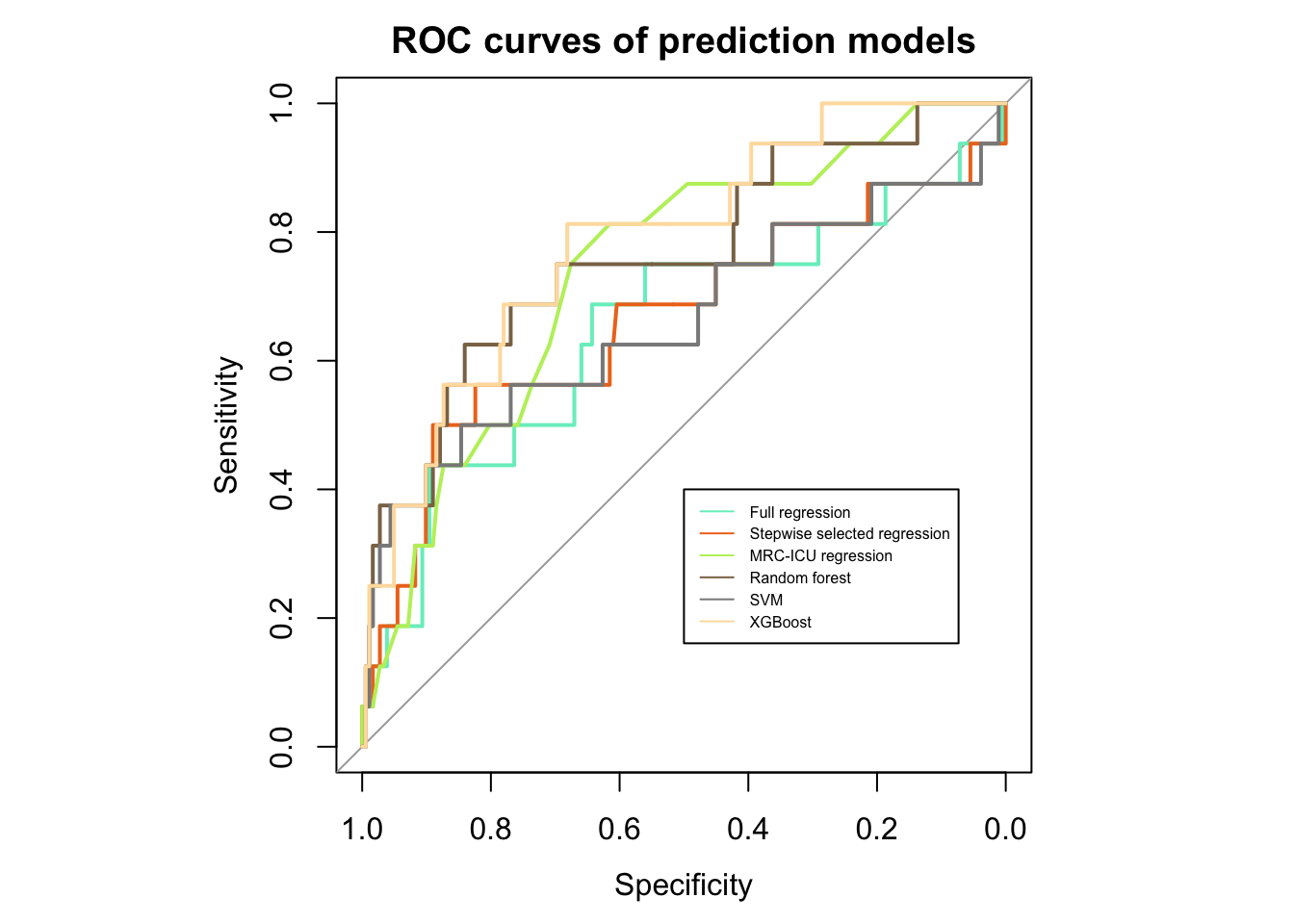


**Supplemental Figure 3.** Most common features for presence of fluid overload prediction with Random Forest imputations


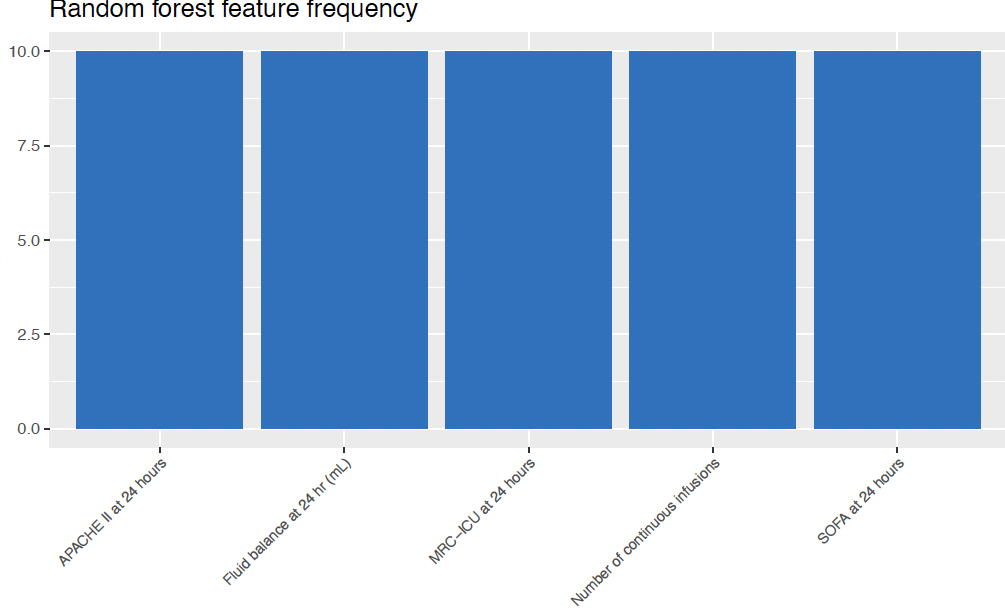


**Supplemental Figure 4.** Most common features for presence of fluid overload prediction with Support Vector Machine imputations


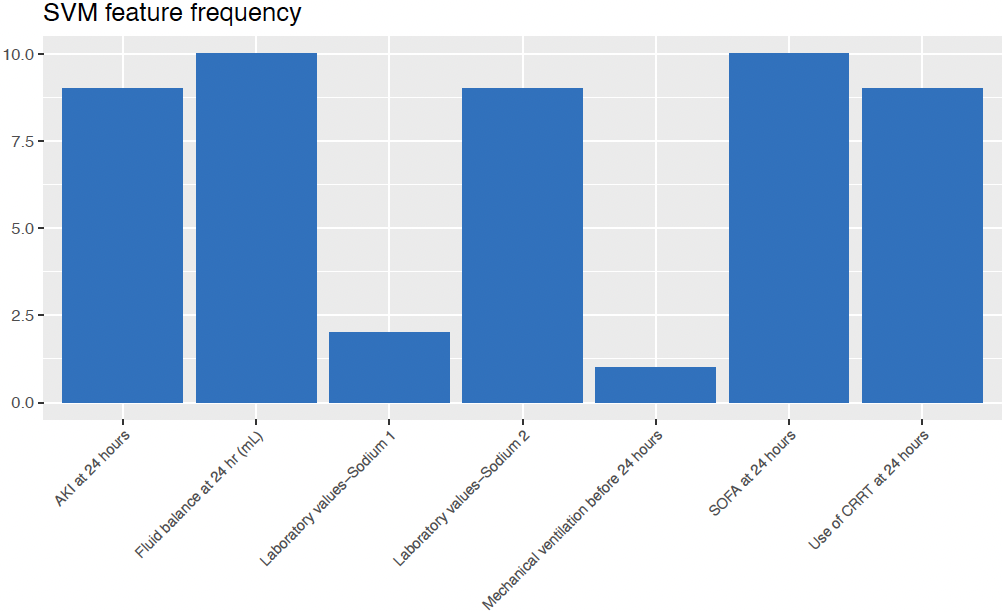


**Supplemental Figure 5.** Most common features for amount of fluid overload prediction with XGBoost imputations


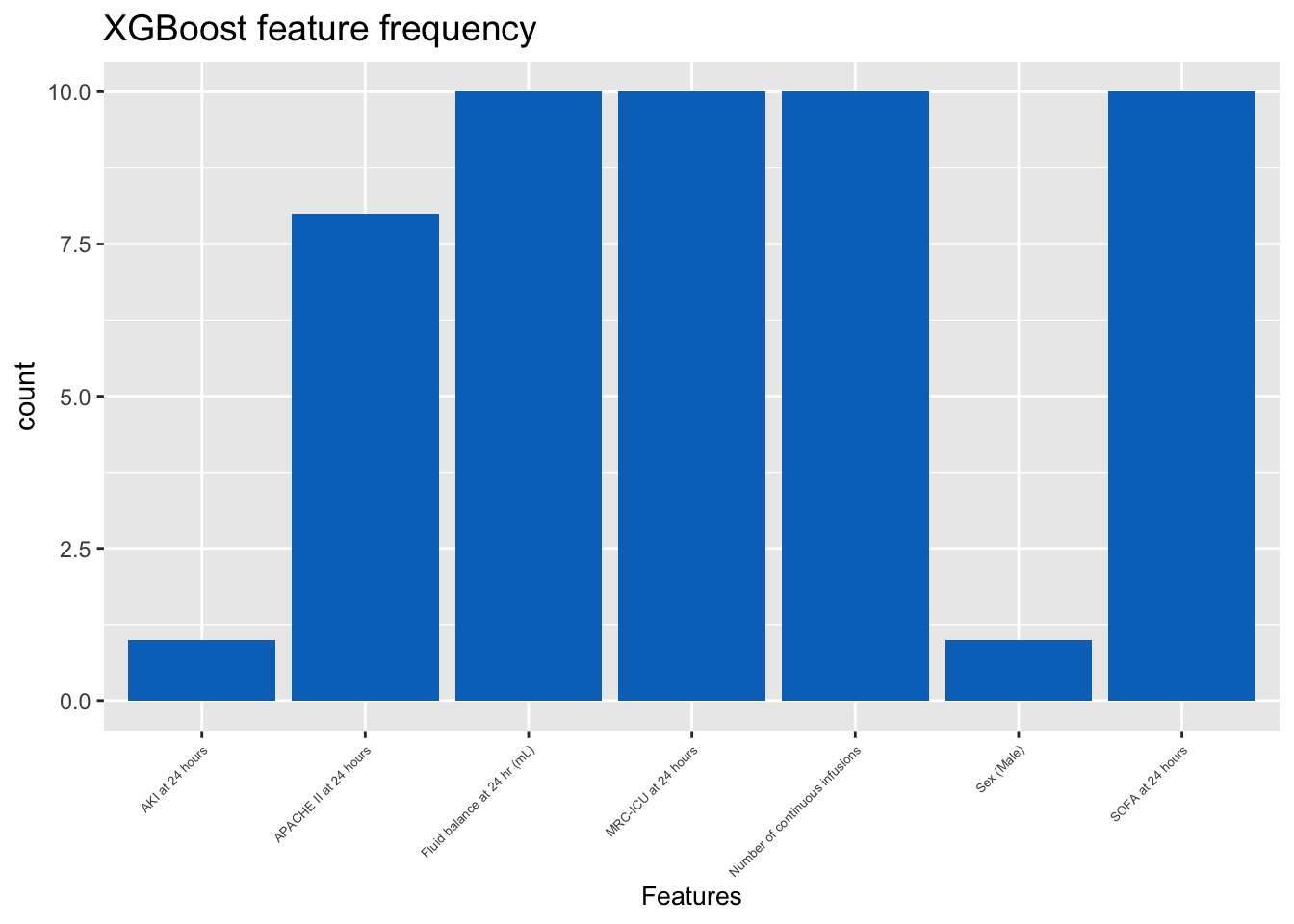


**Supplemental Figure 6.** Most common features for amount of fluid overload prediction with Random Forest imputations


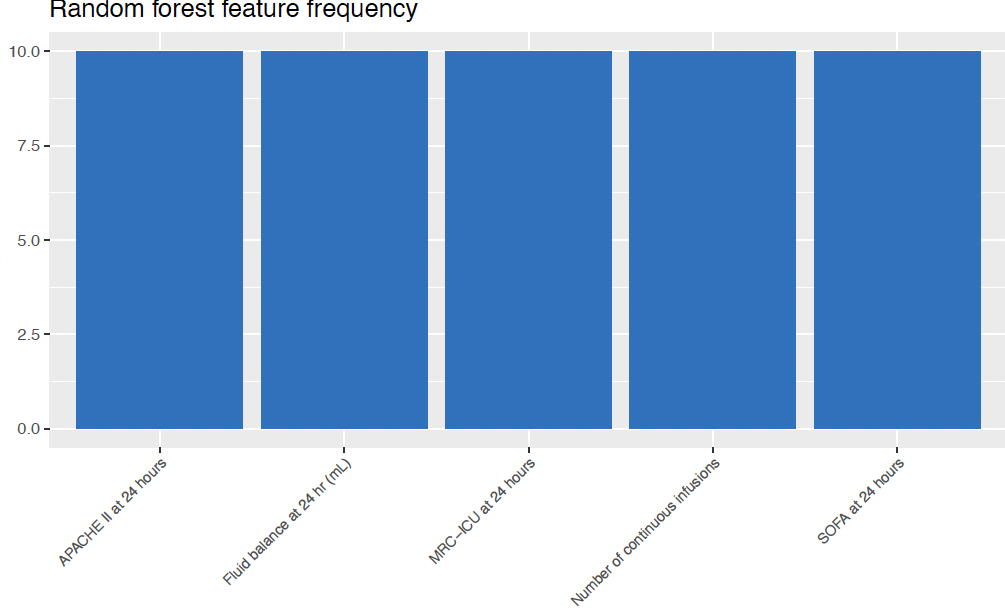


**Supplemental Figure 7.** Most common features for amount of fluid overload prediction with Support Vector Machine imputations


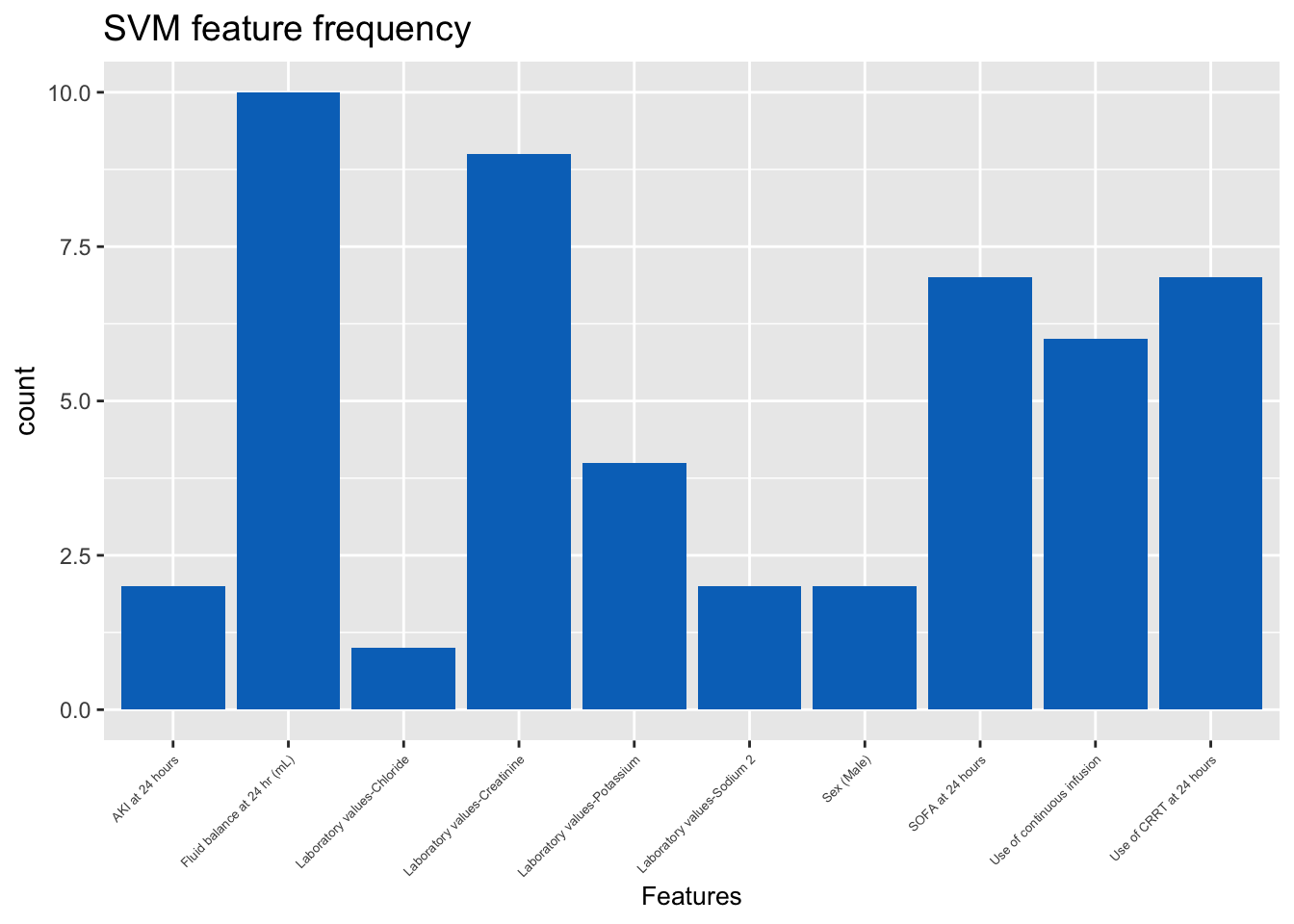

Supplement: Supplementary file 1 — Supplementary Information. [file 41598_2023_46735_MOESM1_ESM.docx]
